# RNAi-mediated silencing of *Trichinella spiralis* serpin-type serine protease inhibitors results in a reduction in larval infectivity

**DOI:** 10.1186/s13567-020-00860-3

**Published:** 2020-11-23

**Authors:** Nana Yi, Pengcheng Yu, Lijia Wu, Zhaokun Liu, Jingzhe Guan, Chang Liu, Mingxu Liu, Yixin Lu

**Affiliations:** Laboratory of Animal Common Disease Prevention, College of Veterinary Medicine, Northeast Agricultural University, 59 Mucai Street, Harbin, 150030 China

**Keywords:** *Trichinella spiralis*, serpin-type serine protease inhibitors (TsSPIs), RNAi, invasion, infectivity

## Abstract

*Trichinella spiralis* serpin-type serine protease inhibitors (TsSPIs) are expressed in adult worms (AW), newborn larvae (NBL) and muscle larvae (ML) of *T. spiralis*, with the ML stage demonstrating the highest expression level. This study aims to determine TsSPI functions in larval viability and invasion of intestinal epithelial cells in vitro, as well as their development, survival, and fecundity in vivo via RNAi. TsSPI-specific siRNAs and dsRNA were transfected into ML by incubation. The silencing effect of TsSPI transcription and expression was determined using qPCR and western blot, respectively. After incubation in 60 ng/μL dsRNA–TsSPI for 3 days, larval TsSPI mRNA and protein expression levels were reduced by 68.7% and 68.4% (P < 0.05), respectively. dsRNA-mediated silencing of TsSPI significantly impacted larval invasion into intestinal epithelial cells in vitro but did not affect the survival rate of larvae. After challenge with dsRNA–TsSPI-treated ML, mice exhibited a 56.0% reduction in intestinal AW burden and 56.9% reduction in ML burden (P < 0.05), but NBL production of female AW remained the same (P > 0.05). Our results revealed that RNAi-mediated silencing of TsSPI expression in *T. spiralis* significantly reduced larval infectivity and survival in the host but had no effect on the survival rate and fecundity. Furthermore, TsSPIs have no effect on the growth and reproduction of parasites but may be directly involved in regulating the interaction of *T. spiralis* and the host. Therefore, TsSPIs are crucial in the process of *T. spiralis* larval invasion and parasite survival in the host.

## Introduction

Trichinellosis is a widespread food-borne parasitic zoonosis whose route of infection is through the ingestion of raw or undercooked meat containing infective muscle larvae (ML) of *Trichinella* [1]. Approximately 11 million people have been infected by this nematode worldwide [2, 3]. Trichinellosis is considered an emerging/re-emerging disease [4]. Trichinellosis is not under control in endemic areas because this disease is widely distributed worldwide [5]. Thus, identifying the *Trichinella spiralis* (*T. spiralis*) invasion-related protein is necessary to prevent the occurrence of trichinellosis.

*Trichinella spiralis* is an intestinal nematode that infects more than 150 mammalian species [6]. The different developmental phases of *T. spiralis* occur in a single host. When contaminated meat is ingested, the encapsulation of *T. spiralis* ML is broken down by gastric juice, ML is liberated in the host’s stomach, and bile activates ML to change to intestinal infectious larvae (IIL). IIL penetrate the intestinal epithelium and mature into adult worms (AW) after four moults. AWs deposit newborn larvae (NBL). NBL enter the blood system, invade skeletal muscle, and develop into ML to complete the life cycle [1, 7]. The evading mechanism has attracted considerable attention from researchers because *T. spiralis* can escape the attack of digestive enzymes and the immune system and successfully parasitize and minimize host damage [8]. *T. spiralis* serine protease inhibitors (TsSPIs), which are the major regulatory antigen in the process of *T. spiralis* host invasion, can inhibit a variety of digestive and immune enzymes of the host [9]. Therefore, investigating the function of TsSPIs is important.

Serine protease inhibitors (SPIs) are a commonly investigated structurally conserved protein superfamily [10]. SPIs are divided into at least 18 families, such as serpin (SPI), Kazal (KaSPI), Kunitz, TAP, and TIL, according to the primary sequence, structural motifs and binding mechanism. Serpin is the most important serine protease inhibitor family [11]. SPIs can control endogenous and exogenous proteolytic activities by inhibiting one or more serine proteases and play important roles in the development, survival, reproduction, inflammation, and apoptosis of animals, plants, parasites, insects and viruses [12, 13]. SPIs are involved not only in the growth and development of parasites, inhibition of blood coagulation, and regulation and suppression of the host immune response [14, 15] but also in the early development stage of parasites in the host. Earlier studies showed that TsSPIs can protect parasites from host digestive enzymes by inhibiting their activity [9, 16]. Thus, TsSPIs may be an important factor in regulating the interaction between *T. spiralis* and the host.

RNA interference (RNAi) was first discovered in the nematode worm *Caenorhabditis elegan*s and has since been widely used to downregulate target molecules [17]. Posttranscriptional gene silencing is a method used to control the reading of mRNA genetic information in organisms. Double-stranded RNA (dsRNA) is degraded into small interfering RNA (siRNA) when dsRNA is transfected into the organism, and then siRNA is guided to the RNA-induced silencing complex (RISC) with Dicer, Argonaute, and RNA-dependent RNA polymerase. siRNA can recognize complementary target mRNA, the antisense siRNA strand in RISC directs binding to corresponding sites of mRNA, and mRNA is degraded by ribonuclease III in RISC [18]. The RNAi technique has been used to investigate the biological function of specific key genes in various helminths, including cathepsin L and Z-like cysteine proteases in *Onchocerca volvulus* [19], pyrophosphatase in *Ascaris suum* [20], beta-tubulin in *Haemonchus contortus* [21], type V collagen in *Schistosoma japonicum* [22], enolase in *Clonorchis sinensis* [23], and paramyosin and Nudix hydrolase in *T. spiralis* [24, 25].

This study aimed to assess the biological roles of TsSPIs in *T. spiralis* viability, invasion, development and reproduction via RNAi. TsSPI-specific siRNA or dsRNA was designed to silence TsSPIs in *T. spiralis* larvae. TsSPI mRNA and protein expression was analysed to determine the silencing effect. The invasive ability, development and survival of dsRNA-treated larvae in mice were observed in this study.

## Materials and methods

### Parasites and experimental animals

The *T. spiralis* T1 strain (ISS3) used in this study was obtained from the Department of Parasitology of Northeast Agricultural University, and its host is a Heilongjiang Xunke pig. BABL/c mice (male, 15–20 g) aged 6–8 weeks were obtained from the Animal Center of Harbin Medical University. Experiments were approved by the Animal Ethics Committee of Harbin Medical University and performed in accordance with animal ethics guidelines and approved protocols (Animal Ethics Committee approval number SYXK [Hei] 2016–007).

### Preparation of siRNA

Full-length cDNA encoding TsSPIs (GenBank accession EU263307.1) was utilized to design siRNA sequences using siDirect version 2.0 [26]. The TsSPI-specific siRNA oligos (Stealth™ RNAi duplexes) used in this work were chemically synthesized by GenePharma (Shanghai, China). Sequences of the three specific siRNAs and control siRNAs used in this study are listed in Table 1. The same control FAM-labelled siRNA (GenePharma, China) was used to evaluate the transfection efficiency.

**Table 1:** The siRNAs used in this study

siRNA name	Sense (5′–3′)	Antisense (5′–3′)
siRNA153	GCUGAAUGUGAUGUUCAAATT	UUUGAACAUCACAUUCAGCTT
siRNA479	CCGUCAACGCAAUUUAUUUTT	AAAUAAAUUGCGUUGACGGTT
siRNA986	CCGAUCGCAUAGUACCCAUTT	AUGGGUACUAUGCGAUCGGTT
Control siRNA	UUCUCCGAACGUGUCACGUTT	ACGUGACACGUUCGGAGAATT

### Synthesis of dsRNA

The target DNA fragment of TsSPIs was generated with PCR using TsSPI-specific primers flanked by T7 RNA polymerase promoter sequences based on the sequence regions 247-8121 in dsRNA–TsSPI transcription (Table 2). In addition, a DNA fragment of GFP was generated with PCR for use as a negative control.

**Table 2:** Gene-specific primers flanked by T7 RNA polymerase promoter sequences

Primer	Sense (5′–3′)
TsSPI dsRNA-566 F	GGTCGTTCGTTTCATCGC
TsSPI dsRNA-566 R	AGCACCGCTAACTTTGGA
TsSPI dsRNA-566 T7 F	TAATACGACTCACTATAGGG GGTCGTTCGTTTCATCGC
TsSPI dsRNA-566 T7 R	TAATACGACTCACTATAGGG AGCACCGCTAACTTTGGA
dsGFP F	TCCTGGTCGAGCTGGACGG
dsGFP T7 F	TAATACGACTCACTATAGGG TCCTGGTCGAGCTGGACGG
dsGFP R	CGCTTCTCGTTGGGGTCTTTG
dsGFP T7 R	TAATACGACTCACTATAGGG CGCTTCTCGTTGGGGTCTTTG

The underlined portion is the T7 promoter sequence

PCR products were purified using a 1% agarose gel and then used to synthesize dsRNA-TsSPI through in vitro transcription using a T7 High Yield RNA Transcription Kit (Vazyme, China). The dsRNAs were visualized on a 1% agarose gel to confirm integrity. The concentration of each dsRNA was determined using a NanoDrop 2000 spectrophotometer (Thermo Scientific, US).

### SiRNA or dsRNA Delivery to T. spiralis Worms

*Trichinella spiralis* ML were recovered from muscles of infected mice, which were incubated at least 40 days postinfection by digestion with an artificial solution of 1% pepsin and 1% HCl at 37 °C for 3 h and washed three times in 0.9% saline solution [27, 28]. Incubation methods were used to deliver specific or control siRNA or dsRNA into the larvae. A total of 5,000 ML were suspended in a final volume of 500 ml RPMI 1640 culture medium (HyClone, US) and supplemented with 100 units/mL penicillin and 100 mg/mL streptomycin for incubation. Control siRNA or dsRNA or specific siRNA–TsSPI or dsRNA–TsSPI were incubated with 2 mL Lipofectamine 2000 Reagent (Invitrogen, US) for 20 min before being added to the larvae to a final concentration of 2 μM for siRNA and 40 ng/μL for dsRNA. The incubation continued at 37 °C and 5% CO_2_ for 7 days. FAM-labelled control siRNA was used to visualize the uptake of siRNA.

Incubation conditions kept the worms alive for 7 days, when control siRNA or dsRNA were added.

### RNA extraction and qPCR analysis of TsSPI mRNA expression

Total RNA was extracted from siRNA- or dsRNA-treated ML 1–6 days posttreatment with control siRNA or dsRNA or 20, 40, 60 and 80 ng/μL dsRNA–TsSPI or 1, 2 and 3 μM siRNA-986 using TRIzol reagent (Invitrogen, US) according to the manufacturer’s instructions. RNA was visualized with 1% agarose gel electrophoresis. RNA concentration was measured with a NanoDrop2000 (Thermo Scientific, US). Total RNA was reverse transcribed to first-strand cDNA using HiScript II Q RT SuperMix for qPCR (+gDNAwiper) (Vazyme, China). The following primers were designed for qPCR: TsSPI (forward: AAGGCAATGCGGTCGTT; reverse: CGGCGATAAGGCGAGTA); *T. spiralis* Kazal-type serine protease inhibitor (TsKaSPI), which is a gene with a similar function of verifying the specificity of dsRNA–TsSPI (forward: GATGGATTCTGCTGCCAAG; reverse: CCAAACAACACATTGCTCGT); and GAPDH, which serves as the housekeeping gene (forward: TGGCTTAGCTCCGTTGG; reverse: TTTGGGTTGCCGTTGTA). qPCR was conducted in triplicate using ChamQ Universal SYBR qPCR Master Mix (Vazyme, China) and a 7500 system (ABI, US) to evaluate target gene expression.

Reactions were performed with 40 cycles at 10 s and 95 °C, as well as 30 s and 60 °C. TsSPI transcription levels in RNAi-treated larvae were calculated as the percentage relative to the level of untreated larvae. Transcription levels of the target gene in siRNA- or dsRNA-treated worms were normalized by subtracting the expression level of GAPDH and then calculating the quantitative data using the comparative Ct (2^−ΔΔCt^) method.

### Western blot analysis of TsSPI protein expression

SiRNA- or dsRNA-treated worms were harvested after 3 days of incubation to observe the effect of siRNA or dsRNA on TsSPI protein expression. Harvested worms were homogenized with liquid nitrogen. RIPA solution was added to the worm fragments (Thermo Scientific, US) and then further homogenized with an ultrasonic disruptor (Scientz, China) (3-s cycle 1 min, 40% power). The supernatant containing soluble crude protein was collected after centrifugation at 5000 rpm for 10 min at 4 °C. The total protein concentrations were determined with a BCA assay (Wanleibio, China). An equal amount of protein from each treated larval group was separated via SDS-PAGE and subsequently transferred onto nitrocellulose membranes. Membranes were cut into strips, blocked with 5% (W/V) skim milk in Tris-buffered saline with 0.05% Tween 20 (TBST), and incubated with mouse anti-TsSPI serum (1:200) or TsKaSPI (1:100) at 37 °C for 2 h. In addition, a mouse antibody against GAPDH (1:1,000) (Proteintech, US) was used to detect GAPDH expression as a quantitative protein control. HRP-conjugated goat anti-mouse IgG (1:8,000) was used as the secondary antibody. After washing, strips were treated using an enhanced chemiluminescence (ECL) kit (CWBIO, China).

### *Viability of ML treated with dsRNA–TsSPI *in vitro

Larvae treated with dsRNA were cultured in 1640 medium at 37 °C and 5% CO_2_ for 6 days. After incubation for 24 h, the viability of ML was observed under an inverted microscope (Nikon, Japan). Larvae without activity and straight as “C” shapes were considered nonviable but counted as dead with continued inactivity for the next 6 h at 37 °C. The activity of living ML was characterized by a wriggling motion [29]. The results expressed the number of live larvae within each experiment. The viability of dsRNA-treated larvae was compared with that of control dsRNA-treated and untreated larvae. Each group was independently analysed five times.

### *Ability of TsSPI-silenced ML to invade intestinal epithelial cells *in vitro

ML were soaked using 60 ng/μl dsRNA–TsSPI or control dsRNA or untreated and cultured for 3 days to assess the effect of TsSPI silencing on the ability of larvae to invade intestinal epithelium cells (Caco-2). ML were then activated into IIL using 5% goat bile at 37 °C for 2 h [30]. Caco-2 cells were grown to confluence in 24-well plates. Each cell monolayer was overlaid with 100 IIL suspended in 0.5 mL of semisolid medium (RPMI 1640 [HyClone, US] containing 15 mM HEPES and 1.75% agarose) [31]. The 24-well plate was incubated at 37 °C in 5% CO_2_ for 2 h. IIL invasion into Caco-2 cells was observed under an inverted microscope (Nikon, Japan). The number of penetrating and motile worms in intestinal epithelial cells (IECs) was counted, whereas worms that were still suspended or coiled in semisolid medium were considered nonpenetrated worms [32]. Each group was independently analysed five times.

### Development and survival of TsSPI-silenced larvae in mice

Sixty mice were equally divided into three groups to examine the infectivity of TsSPI-silenced larvae. The three groups were orally inoculated with 300 T*. spiralis* ML soaked in PBS, control dsRNA, and dsRNA–TsSPI. Ten mice from each group were sacrificed 4 days postinfection (dpi), and AWs were collected from the intestines of sacrificed mice.

The fecundity of recovered female worms was observed after individual incubation in each well of the 24-well plate with culture medium (RPMI 1640 containing 10% heat-inactivated foetal bovine serum, 100 U penicillin/mL and 100 mg streptomycin/mL) at 37 °C in 5% CO_2_ for 72 h, and the number of NBL produced by each female worm was counted [33]. ML were collected from the 10 remaining mice in each group at 40 dpi by artificial digestion, as previously described. The parasite burden reduction was calculated on the basis of the mean number of intestinal AW and larvae per gram (LPG) of muscles recovered from the dsRNA–TsSPI group compared with the PBS or control dsRNA group [34, 35].

### Statistical analysis

Statistical analyses were performed using GraphPad Prism 5 expressed as the mean ± standard deviation (SD). Differences in the relative expression of mRNA and protein, worm burden and fecundity among the groups were analysed using one-way ANOVA. Statistical significance was defined by a P value of < 0.05.

## Results

### SiRNA delivery to T. spiralis ML

**Figure 1:**
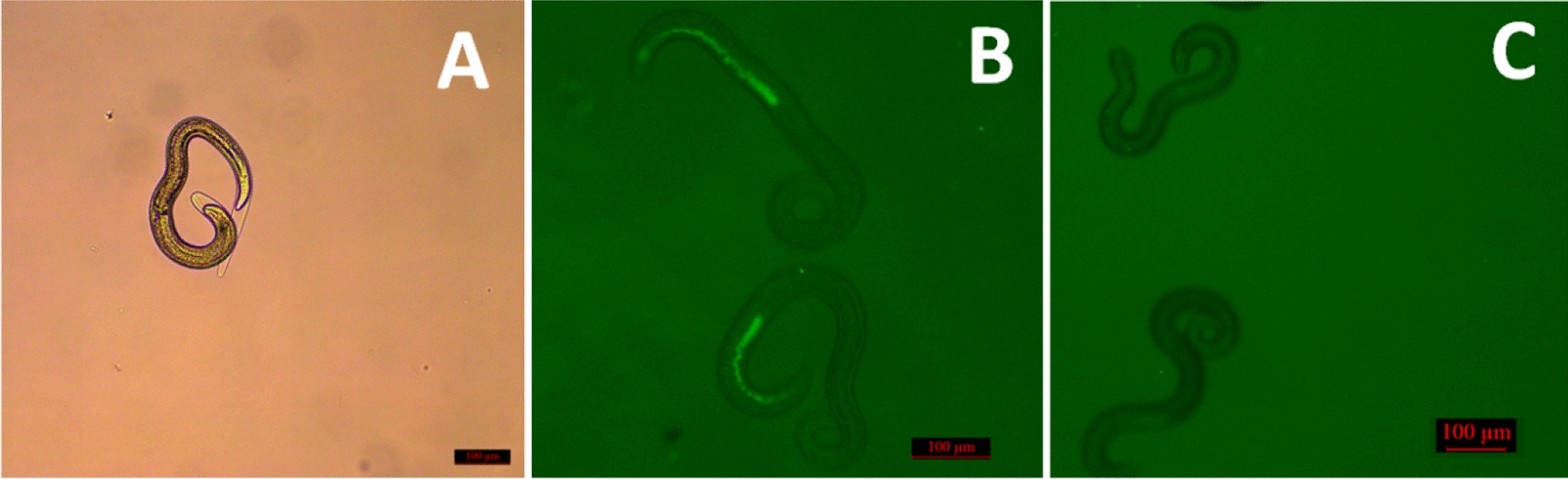
**Detection of siRNA delivery to T. spiralis larvae via fluorescence microscopy.** Larvae were transfected with FAM-labelled control siRNA by incubation. **A** Some larvae begin to moult after incubation for 12 h. **B** Uptake of FAM-labelled siRNA into larvae under the fluorescence microscope. **C** Absence of fluorescence in untreated larvae.

Some ML began to moult after incubation with FAM-labelled control siRNA for 12 h (Figure 1A). Fluorescence staining was observed in ML under fluorescence microscopy (Figure 1B). However, the absence of fluorescence staining in untreated larvae (Figure 1C) demonstrated that siRNA can be efficiently delivered into *T. spiralis* larvae through incubation.

### Specific siRNA- or dsRNA-mediated suppression of TsSPI mRNA expression

**Figure 2:**
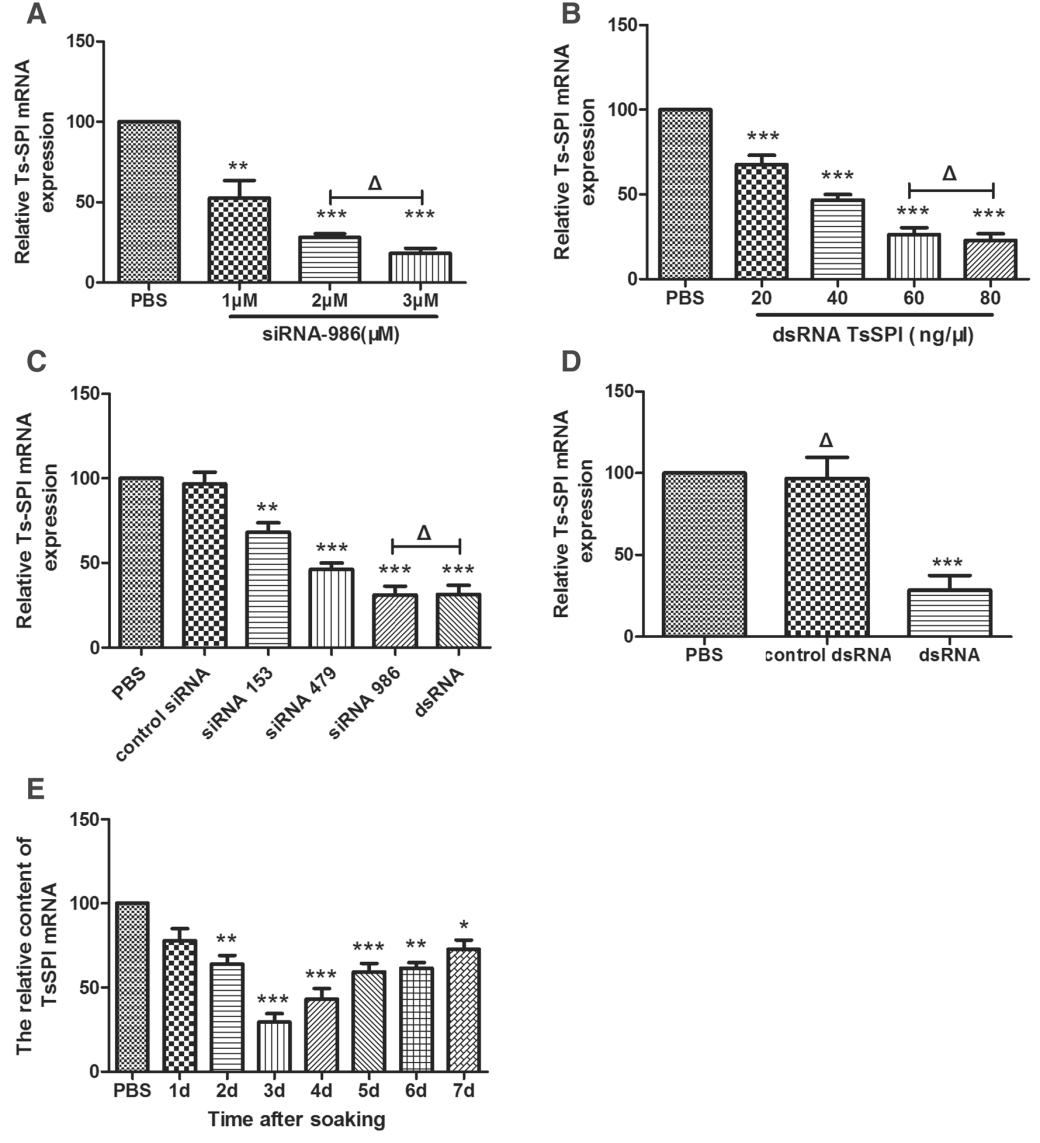
**qPCR analysis of TsSPI mRNA expression mediated by siRNA and dsRNA.** Relative transcription levels of TsSPI in larvae after **A** incubation for 3 days with various siRNA-986 concentrations, **B** incubation for 3 days with various dsRNA concentrations, **C** incubation for 3 days with various siRNAs and dsRNA, **D** incubation for 3 days with control dsRNA and dsRNA, and **E** incubation for a longer period. Assays were performed in triplicate, and the data are expressed as the mean ± SD. *P < 0.05, **P < 0.01, ***P < 0.001, and **ΔP** > 0.05 compared with the PBS group.

The qPCR results showed that the TsSPI gene was 52.6%, 28.1%, and 18.2% of the relative transcription level compared with the control group in ML treated with 1, 2 and 3 μM siRNA-986, respectively (P < 0.05) (Figure 2A). After incubation with 20, 40, 60 and 80 ng/μL dsRNA–TsSPI for 3 days, the relative transcription levels of the TsSPI gene in treated ML were 67.6%, 46.5%, 26.4% and 22.7% of the relative transcription level compared with untreated ML, respectively (P < 0.05) (Figure 2B). The efficacy of siRNA and dsRNA silencing was dose-dependent, although the silencing efficiency achieved with 3 μM siRNA or 80 ng/μL dsRNA was the optimal dose, and the difference between 2 and 3 μM siRNA or 60 and 80 ng/μL dsRNA–TsSPI was not statistically significant (P > 0.05). Therefore, 2 μM siRNA and 60 ng/μL dsRNA–TsSPI were used to optimize the working concentration.

When 2 μM siRNA-153, siRNA-479, siRNA-986 or 60 ng/μL dsRNA–TsSPI was transfected into ML for 3 days, the TsSPI gene expression levels in treated larvae were 68.0%, 46.0%, 31.3%, or 31.6% (P < 0.05) of the relative transcription level compared with untreated larvae, respectively (Figure 2C). The expression level of *T. spiralis* ML was significantly reduced in TsSPI mRNA compared with untreated worms. However, both control siRNA (Figure 2C) and control dsRNA (Figure 2D) clearly had no inhibitory effect on TsSPI gene transcription compared with the PBS group (P > 0.05).

After incubation with 60 ng/μL dsRNA–TsSPI for 1, 2, 3, 4, 5, 6, and 7 days, the TsSPI gene in treated larvae was 77.9%, 63.9%, 29.4%, 43.2%, 59.4%, 61.5% and 72.8% of the relative transcription level (P < 0.05) compared with untreated larvae, respectively (Figure 2E).

### Specific siRNA- or dsRNA-mediated suppression of TsSPI protein expression

**Figure 3:**
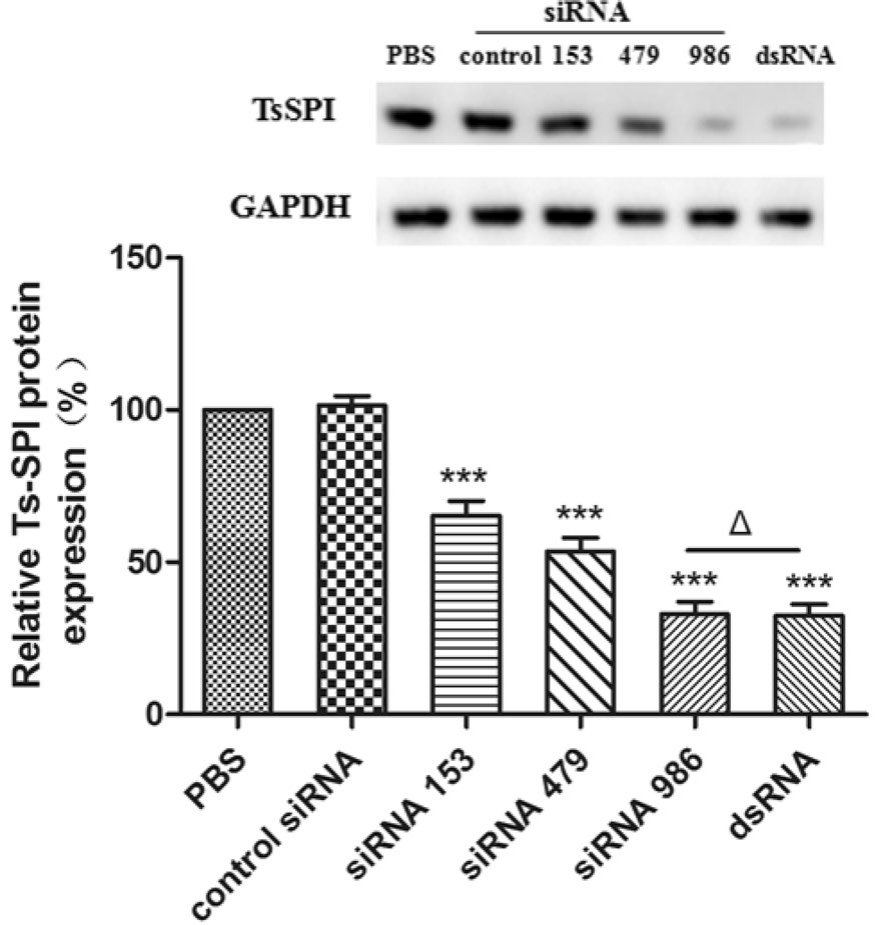
**Western blot analysis of TsSPI protein expression mediated by siRNA and dsRNA.** Western blot with anti-TsSPI-specific antibodies showing the specific inhibition of TsSPI protein expression in crude somatic extracts of *T. spiralis* larvae treated with different siRNAs and dsRNA for 3 days. Assays were performed in triplicate, and the data are expressed as the mean ± SD. *P < 0.05, **P < 0.01, ***P < 0.001, and **ΔP** > 0.05 compared with the PBS group.

**Figure 4:**
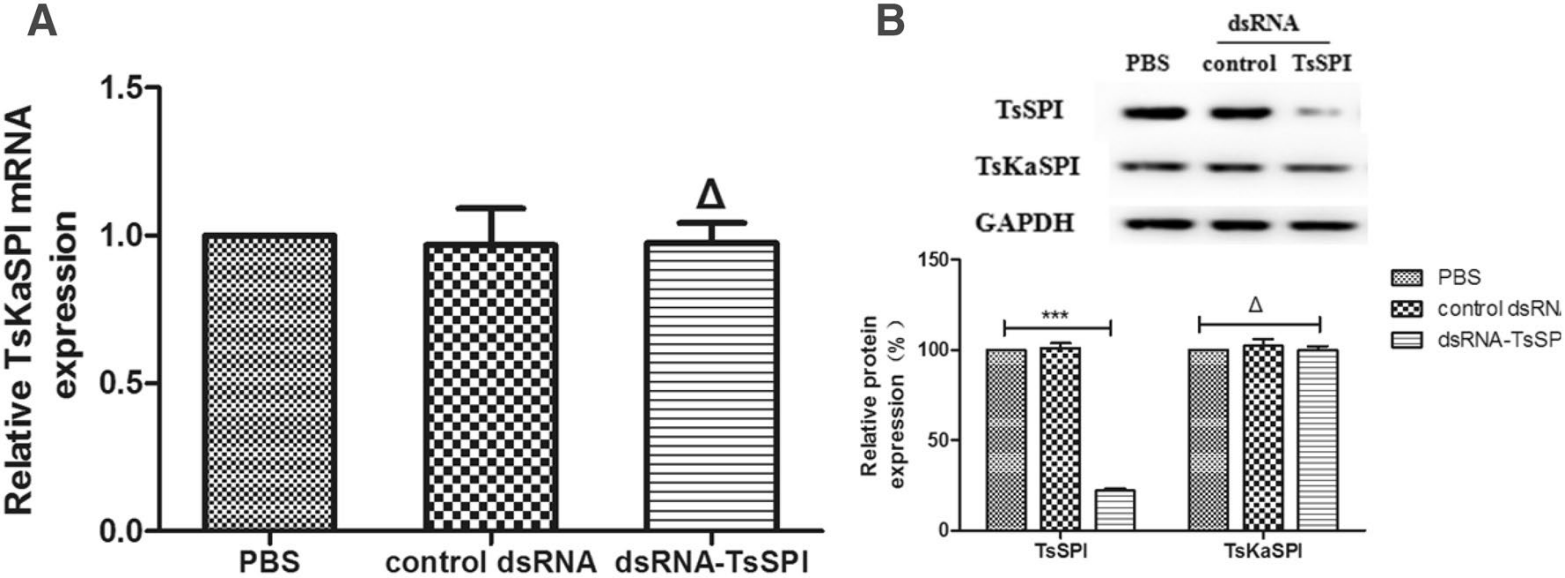
**Gene specificity of RNAi induced by dsRNA–TsSPI. A** qPCR analysis of relative transcription levels of the TsKaSPI gene in larvae after incubation for 3 days in dsRNA–TsSPI. **B** Western blot analysis of anti-TsSPI- and anti-TsKaSPI- specific antibodies of relative TsSPI and TsKaSPI protein expression levels after incubation in dsRNA–TsSPI for 3 days. Assays were performed in triplicate, and the data are expressed as the mean ± SD. *P < 0.05, **P < 0.01, ***P < 0.001, and **ΔP** > 0.05 compared with the PBS group.

When ML was incubated in 2 μM siRNA-153, siRNA-479, siRNA-986 or 60 ng/μL dsRNA–TsSPI for 3 days, the expression levels of the TsSPI protein were inhibited by 64.5%, 53.6%, 32.9% and 32.4% (P < 0.05), respectively, compared with the PBS group (Figure 3). However, both the control siRNA (Figure 3) and control dsRNA (Figure 4B) clearly had no inhibitory effect on the expression levels of TsSPI protein compared with the PBS group (P > 0.05).

SiRNA–986 and dsRNA–TsSPI demonstrated the maximum silencing of TsSPI mRNA or protein expression, and no significant difference existed between siRNA-986 and dsRNA–TsSPI (P > 0.05). Therefore, 60 ng/μl dsRNA–TsSPI was used to optimize the working concentration for subsequent experiments.

### Gene specificity of RNAi induced by dsRNA–TsSPI

dsRNA-TsSPI (60 ng/μl) was transfected into ML by incubating for 3 days to determine the gene specificity of RNAi induced by dsRNA–TsSPI. The qPCR results showed that the dsRNA–TsSPI–treated group clearly demonstrated no inhibitory effect on the expression levels of TsKaSPI mRNA compared with the PBS group (P > 0.05) (Figure 4A). Western blot analysis showed that the relative protein expression level of TsSPI was clearly reduced in larvae soaked in dsRNA–TsSPI for 3 days compared with untreated larvae (P < 0.05), but the expression of TsKaSPI protein had no inhibitory effect compared with the PBS group (P > 0.05) (Figure 4B).

### *Effect of dsRNA–TsSPI on ML viability *in vitro

**Figure 5:**
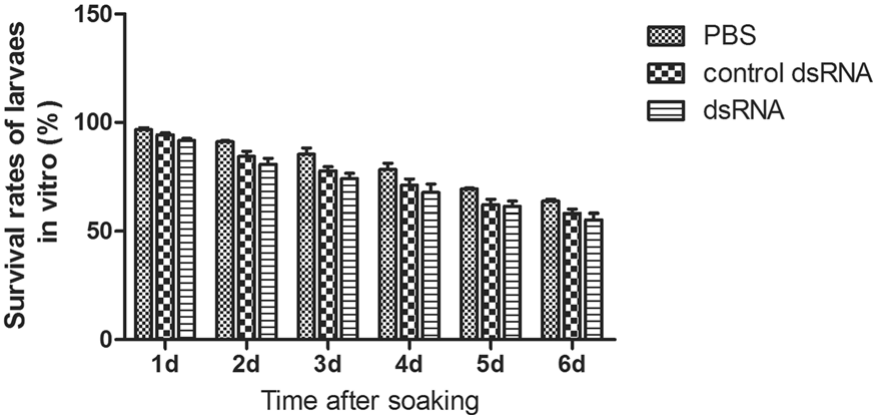
**Effect of dsRNA–TsSPI on ML viability in vitro.** ML was soaked in control dsRNA or dsRNA–TsSPI for 6 days. The number of live larvae, which were determined to be active by wriggling movements, was counted.

When ML was soaked in control dsRNA or dsRNA**–**TsSPI for 6 days, the difference was not statistically significant (P > 0.05), although the dsRNA–TsSPI-treated group demonstrated a lower survival rate of larvae than the PBS or control dsRNA groups (Figure 5).

### Inhibition of larval invasion into intestinal epithelial cells by dsRNA-mediated silencing of TsSPIs

**Figure 6:**
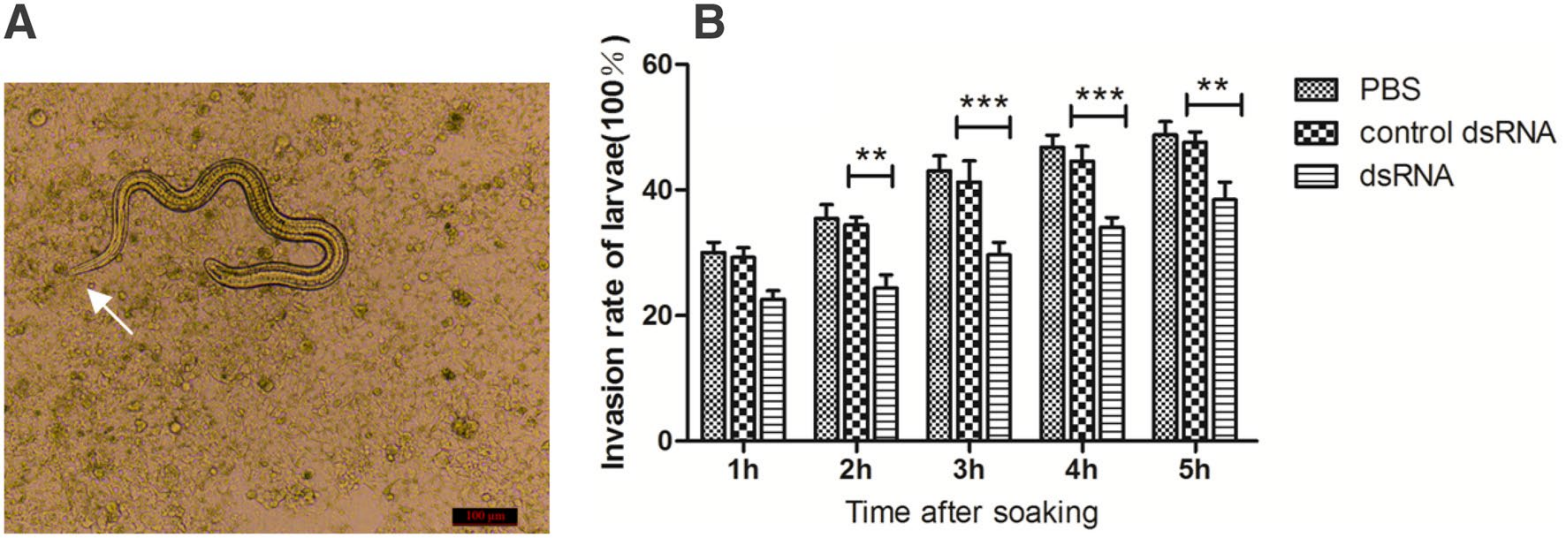
**Effect of dsRNA–TsSPI on the capacity of larvae to invade IECs in vitro. A** IIL larvae invading Caco-2 and migrating in the monolayer. **B** Invasion rate of larvae during a prolonged period after incubation. Assays were performed in triplicate, and the data are expressed as the mean ± SD. *P < 0.05, **P < 0.01, ***P < 0.001, and **ΔP** > 0.05 compared with the control dsRNA group.

When IIL was added to Caco-2 cells and cultured for 2 h, the IIL intruded into Caco-2 cells and migrated in a monolayer (Figure 6A). The dsRNA-mediated silencing of TsSPIs significantly suppressed larval invasion into the Caco-2 monolayer compared with the control dsRNA group (P < 0.05). However, the difference in inhibiting the invasion of Caco-2 by the larvae soaked in control dsRNA and untreated larvae was not statistically significant (P > 0.05) (Figure 6B).

### *Effect of the *in vivo* larval infectivity, development, and fecundity *via* dsRNA-mediated silencing of TsSPI*

**Figure 7:**
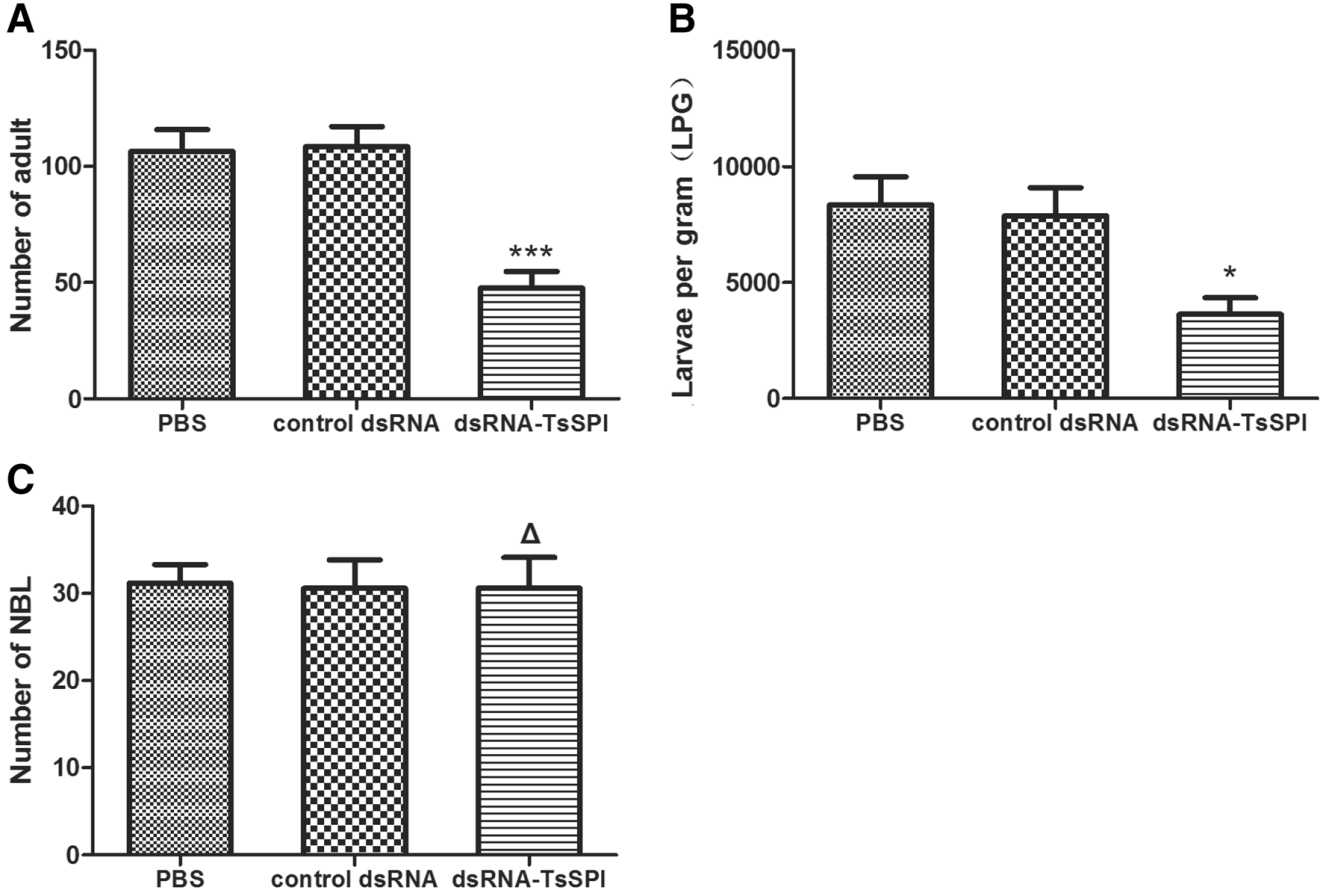
**A Number of AWs, B LPG of muscles, and C number of NBL recovered from mice infected with larvae transfected with dsRNA–TsSPI.** Assays were performed in triplicate, and the data are expressed as the mean ± SD. *P < 0.05, **P < 0.01, ***P < 0.001, and **ΔP** > 0.05 compared with the control dsRNA group.

Mice inoculated with *T. spiralis* larvae transfected with dsRNA–TsSPI showed statistically significant reductions of 56.0% and 53.9% in intestinal AW and ML burden (P < 0.05), respectively, compared with the control dsRNA group. There was no significant reduction in AW and ML burden in mice inoculated with ML transfected using control dsRNA compared with mice inoculated with untreated larvae (Figure 7A, B).

The NBL production of female AW from mice inoculated with larvae soaked in dsRNA–TsSPI did not show a significant reduction compared with the control dsRNA group after culturing in vitro for 72 h (P > 0.05) (Figure 7C).

The results suggested that dsRNA-mediated silencing of TsSPIs significantly impacts larval infectivity and invasion but has no effect on the fecundity of female AW.

## Discussion

Sequencing of the entire genomic *T. spiralis* has been completed, but the functions and biological characteristics of many genes remain unclear [36]. The RNAi technique can effectively investigate *T. spiralis* gene function. RNAi has been widely used in various fields of eukaryotic biology because of its simple operation and strong repeatability in *C. elegans* [37, 38], especially when organisms are unsuitable for classical genetic methods that directly evaluate gene function. The functions of *T. spiralis* paramyosin (Tspmy) in the viability and growth development of *T. spiralis* were confirmed for the first time using silencing transcription of Ts-pmy mRNA with RNAi [24]. The new genome editing technique CRISPR-Cas9 has been widely used in recent years to investigate eukaryotes, including protozoa [39, 40], but it is difficult to perform in multicellular parasites. Therefore, RNAi was used in this study to evaluate the function of TsSPIs in the life cycle of *T. spiralis.* The reduction of mRNA and protein expression levels of TsSPIs by 68.4% and 67.6%, respectively, when dsRNA–TsSPI was effectively delivered into the larvae by incubation indicated that the expression of TsSPI mRNA and protein was significantly inhibited by dsRNA–TsSPI.

In this study, dsRNA-mediated silencing of TsSPIs significantly inhibited IIL invasion into intestinal epithelial cells but did not affect the larval survival rate in vitro. The viability of AW was indirectly affected by the RNAi mediation of ML. Larvae developing into AW must invade the intestinal epithelium [7], but dsRNA-mediated silencing of TsSPIs in ML inhibits larvae penetration into the intestinal epithelium and thus hinders the larvae from maturing to AW and depositing NBL in vivo. When mice were inoculated with larvae transfected with dsRNA–TsSPI, the mice exhibited a 56.0% and 56.9% reduction of intestinal AW and ML burden, respectively, but the NBL production of the same number of female AW remained the same. SPI is a kind of protein superfamily that uses a distinct inhibitory mechanism [10, 41]. In this study, TsSPIs were expressed in AW, NBL, IIL and ML of *T. spiralis* but significantly expressed in ML [9]. Another serpin-type TsSPI that was highly expressed in NBL can affect the development and invasion of *T. spiralis* and plays a key role in female fecundity [31, 42, 43]. Therefore, TsSPIs may play a crucial role in the process of the high expression stage. The TsSPI in this study did not affect the survival and fecundity of *T. spiralis* in vitro but was conducive to ML invasion into intestinal epithelial cells and maturation into AW. The results further demonstrated that TsSPI may not be involved in the growth and reproduction of parasites but is directly involved in regulating the interaction of *T. spiralis* and the host to some extent. Previous studies have shown that TsSPIs can alleviate inflammatory bowel disease, inhibit the host immune response [42, 44, 45], and effectively inhibit the activity of digestion and inflammatory enzymes in the different stages of the *T. spiralis* life cycle and host tissues [9, 46, 47]. TsSPIs can be identified as the major regulatory antigen in the process of *T. spiralis* host invasion by inhibiting the host’s enzyme and immune response.

In conclusion, our results demonstrated that silencing TsSPI expression in *T. spiralis* significantly reduced larval infectivity and survival in the host, and TsSPIs plays an important role during the process of *T. spiralis* larval invasion and survival in the host.

## Data Availability

The datasets used or analysed during the current study are available from the corresponding author on reasonable request.

## Abbreviations

AW: adult worms
dpi: days postinfection
dsRNA: double-stranded RNA
ECL: enhanced chemiluminescence
IECs: intestinal epithelial cells
IIL: intestine infective larvae
LPG: larvae per gram
ML: muscle larvae
NBL: newborn larvae
RISC: RNA-induced silencing complex
RNAi: RNA interference
SD: standard deviation
siRNA: small interfering RNA
SPI: serine protease inhibitor
*T. spiralis*: *Trichinella spiralis*
Tspmy: *T. spiralis* Paramyosin
TsSPI: *T. spiralis* Serine protease inhibitors
